# Electricity in the Suppression of the Lacteal Secretion

**Published:** 1857-11

**Authors:** 


*Electricity in the Suppression of the Lacteal Secretion.*

M. Becquerel, in a late communication to the Societe Medicale des Hopitaux de Paris, has made some remarks upon the influence of electricity in restoring the secretion of milk. His attention was called to the subject by a case related to him by M. Aubert, who had employed electricity in the case of a young woman whose milk had been suppressed in consequence of a double pneumonia. The electricity was applied to the breasts by means of moist excitors, and after four applications, each lasting twenty minutes, the lacteal secretion was completely restored. M. Becquerel was at first incredulous as to the reality of the result; but the following case, which fell under his observation, removed his doubts:

A young woman, aged twenty-seven, well formed, although of a nervous temperament, had suckled a young infant for six months, but, on the occasion of some intense and often-repeated mental emotions, the lacteal secretion diminished considerably; the right breast retained a little milk, but the left was almost completely dried up. M. Becquerel applied the electrical current at first to the left breast, placing the moist excitors, made of sponge, successively in the different points of the circumference of the breast, so that the currents might traverse the organ in all directions. Three applications were made, each lasting a quarter of an hour. The patient suffered very little, and indeed experienced little more than a feeling of inconvenience. From the time of the first application, the rush of milk supervened almost immediately after the application of the electrical currents. After the third application, the secretion was full and entire; the child had taken the breast, and the milk was abundant in the left breast, and sufficient in the right to obviate the necessity of applying the electricity on that side.—*Dublin Hospital Gazette*, July 10, from *L' Union Medicale*, Jan. 3, 1857.

